# Suspected Central Nervous System Infections in HIV-Infected Adults

**DOI:** 10.3389/fneur.2021.741884

**Published:** 2021-09-17

**Authors:** Fereshte Sheybani, Diederik van de Beek, Matthijs C. Brouwer

**Affiliations:** ^1^Department of Neurology, Amsterdam Neuroscience, Amsterdam University Medical Centres (UMC), University of Amsterdam, Amsterdam, Netherlands; ^2^Department of Infectious Diseases and Tropical Medicine, Faculty of Medicine, Mashhad University of Medical Sciences, Mashhad, Iran

**Keywords:** HIV, central nervous system infections, cerebrospinal fluid, CD4 lymphocyte count, progressive multifocal leukoencephalopathy

## Abstract

**Objectives:** To study the differential diagnosis of HIV-infected patients with suspected central nervous system (CNS) infections and the association of CD4 counts with the final diagnosis.

**Methods:** We analyzed HIV-infected patients from a prospective cohort study on the diagnostic accuracy of clinical and laboratory characteristics in adults with suspected CNS infections in an academic hospital in Amsterdam, the Netherlands, who underwent cerebrospinal fluid (CSF) examination.

**Results:** Thirty-four (9.4%) out of 363 patients with suspected CNS infections were HIV-positive of whom 18 (53%) were diagnosed to have CNS infection, with median CD4 counts of 255 cells/μl. The spectrum of CNS infections consisted of progressive multifocal leukoencephalopathy in three patients (17%); cryptococcal meningoencephalitis, toxoplasma encephalitis, angiostrongylus eosinophilic meningitis, and HIV encephalitis each in two (11%); and cytomegalovirus encephalitis, neurosyphilis, tuberculous meningoencephalitis, histoplasma encephalitis, and varicella-zoster virus meningitis each in one (6%). Clinical characteristics and blood parameters did not differ between HIV-infected patients with CNS infections and other diagnoses. The best predictor for CNS infections was the CSF leukocyte count (AUC = 0.77, 95 CI% 0.61–0.94). The diagnosis of CNS infection was not associated with the CD4 count (median 205 vs. 370, *p* = 0.21). Two patients (11%) with CNS infections died and two (11%) had neurological sequelae.

**Conclusions:** Half of the patients with suspected CNS infections are diagnosed with a CNS infection, and this was not related to CD4 counts. The best predictor for CNS infections was the CSF leukocyte count.

## Introduction

Human immunodeficiency virus (HIV) is a neurotrophic, neuroinvasive, and neurovirulent pathogen (1), which can cause direct infection of the central nervous system (CNS) but also predisposes to a variety of other neuroinfections through impaired T-cell mediated immunity (2–4). It has been estimated that in the era before the introduction of combination antiretroviral therapy (cART), around 10% of HIV-infected patients initially presented with neurological disorders, and 30–50% of them developed neurological complications during the progression of their disease (5). Although recent large observational cohorts of HIV-infected patients have reported a significant decrease in the overall incidence of the most frequent HIV-associated neurological disorders in the cART era (6, 7), CNS infections have remained an important cause of morbidity and mortality (8).

Diagnosis of CNS infections in HIV-infected patients can be challenging as a variety of conditions should be considered in the differential diagnosis, and it may be caused by a broad spectrum of pathogens with diverse and overlapping clinical manifestations (9). The cause of CNS infections in HIV have been related to the CD4 lymphocyte count, of which a value below 200 has been described to predispose to cerebral toxoplasmosis, progressive multifocal leukoencephalopathy (PML), and cryptococcal meningitis (10, 11). HIV patients with higher CD4 counts, on the other hand, are expected to be infected with non-opportunistic pathogens (9). A number of studies have reported the spectrum of CNS infections in patients with a low range of CD4 count (7, 12–14), but the distribution of various CNS infections has not addressed in HIV-infected subgroups with a higher range of CD4 count.

We describe a population of HIV-infected patients included in a prospective study on the diagnostic accuracy of clinical and laboratory characteristics in patients with suspected CNS infections. We analyzed the causes of CNS infections, the differential diagnosis, and the association of CD4 counts with the final diagnosis.

## Methods

We studied HIV-positive patients over 16 years included in a cohort of patients with suspected CNS infections in an academic hospital in Amsterdam, the Netherlands, who underwent CSF examination. The methods have been described in detail previously (15). Data collection regarding past medical history, clinical characteristics on presentation, and results of ancillary investigations including blood and CSF analysis, microbiological examinations, and imaging studies was done using online case record forms.

The final diagnosis was classified by two researchers based on all available clinical data, including microbiological examination of CSF. The classification was performed sorting each diagnosis into one of the following five categories: (1) CNS infection, (2) nervous system inflammation without infection, (3) non-infectious non-inflammatory neurological disorder, (4) non-neurological infection, and (5) other systemic disorder. Outcome was scored using the Glasgow outcome score (GOS). A favorable outcome was defined as a score of 5, and an unfavorable outcome was defined as a score of 1–4.

Continuous data were described with medians and interquartile range and categorical variables with frequency and percentage. The Mann–Whitney *U*-test was used for continuous variables, whereas Fisher's exact test and chi-square tests were used for categorical variables, as appropriate. The area under the curve *(*AUC) was measured using the receiver operating characteristic curve (ROC). A *p*-value < 0.05 was considered as statistically significant. The study was approved by the medical ethical committee of the Academic Medical Centre, Amsterdam, the Netherlands.

## Results

Between February 2012 and May 2015, 363 patients with suspected CNS infections were included of whom 34 were HIV-infected (9.4%). Overall, out of 363 cases with suspected CNS infections, 88 (24%) had CNS infection, 38 (11%) non-infectious non-inflammatory neurological disorders, 121 (33%) non-neurological infections, 108 (30%) non-infectious non-neurological disorders, and 8 (2%) non-infectious neuro-inflammatory disorders. The median age of included HIV-infected patients was 44 years, and 24 of 34 (70%) were male. On admission, 19 (55%) patients were on cART, 1 (3%) stopped treatment shortly before admission, and 10 (29%) were not taking cART despite being diagnosed with HIV infection. In four (11%) patients, HIV infection was newly diagnosed during the workup of neurological manifestations.

Presenting signs and symptoms included fever in 10 patients (29%), headache in 16 (47%), neck stiffness in 2 (5%), altered mental status (defined as Glasgow coma scale of <14) in 5 (14%), and focal neurological deficits in 3 (8%). The median CD4 count was 255 [interquartile range (IQR) 92–480]. Out of 34 patients, 15 (44%) had a CD4 count below 200, 11 between 200 and 500 (32%), and 8 above 500 (24%). CSF examination showed an elevated leukocyte count (>5/mm^3^) in 20 of 34 (59%) patients (Table 1).

**Table 1:** Characteristics of 34 HIV-infected patients with suspected central nervous system infection.

**Characteristic**	**CNS infections**	**Other diagnoses**	**Total**
Age, years	44 (37–51)^†^	45 (38–59)	44 (37–53)
Men	13/18 (72)	11/16 (68)	24/34 (70)
New HIV diagnosis	3/18 (17)	1/16 (6)	4/34 (12)
**Other underlying comorbidities**			
Diabetes mellitus	1/18 (5)	3/16 (18)	4/34 (12)
Cancer	1/18 (5)	1/16 (6)	2/34 (6)
Alcoholism	0/18 (0)	3/16 (18)	3/34 (9)
CD4 count, cells/μL	205 (55–455)	370 (150–500)	255 (93– 480)
Antiretroviral therapy	9/17 (50)	10/16 (62)	19/33 (56)
**Clinical manifestations**			
Presentation <24 h	5/18 (28)	2/16 (12)	7/34 (20)
Headache	9/18 (50)	7/16 (43)	16/34 (47)
Fever	4/17 (22)	6/16 (37)	10/33 (30)
Impaired level of consciousness	5/18 (27)	0/16 (0)	5/34 (15)
Neck stiffness	0/18 (0)	2/16 (12)	2/34 (6)
Seizure	2/18 (11)	1/16 (6)	3/34 (9)
Focal neurological deficits	5/18 (28)	2/16 (12)	7 (21)
**Blood parameters**			
Leukocyte count >12,000/μl	2/18 (11)	5/16 (31)	7/34 (21)
CRP, mg/L	5 (1–16)	27 (5–69)	11 (2–34)
**CSF parameters**			
Opening pressure, mm H2O	27 (11–38)	17 (11.5–20.5)	18.5 (11.7–27)
Leukocyte count, cells/μl	78 (6–247)	5 (2–9)	8 (3–107)
Leukocyte count >5/μl	14/18 (78)	6/16 (38)	20/34 (59)
Leukocyte count >100/μl	8 (44)	0 (0)	8 (24)
CSF-blood glucose ratio	0.51 (0.36–0.61)	0.62 (0.55–0.84)	0.56 (0.41–0.67)
Protein concentration, mg/dl	91 (53–157)	44 (30–64)	58 (36–102)
Unfavorable neurological outcome (GOS <5)	4/18 (22)	4/16 (25)	8/34 (23)
Death	2/18 (11)	0/16 (0)	2/34 (6)

*CNS, central nervous system; CRP, C-reactive protein; CSF, cerebrospinal fluid; GOS, Glasgow outcome scale*.

† *Numbers are represented by the number of patients with specific characteristics/the number of patients evaluated (percent) for nominal variables and by median (25th quartile, 75th quartile) for quantitative variables*.

Out of 34 HIV-infected patients, 18 (53%) were categorized as having a CNS infection, 7 (21%) with non-infectious non-inflammatory neurological disorders, 6 (18%) with non-neurological infections, 2 (6%) with non-infectious non-neurological disorders, and 1 (3%) with non-infectious neuro-inflammatory disorders. Of those who were finally diagnosed with CNS infection, 13 (72%) were male and 5 (28%) females with a median age of 43 years. Eight (44%) patients with CNS infections were on cART, and one discontinued treatment 1 month before. Their median CD4 was 205 (IQR 55–455). Out of 18 patients with CNS infection, 4 (22%) had a normal CSF leukocyte count. Only 1 (6%) out of 18 patients with CNS infection and 3 (19%) out of 16 with other diagnoses had completely normal CSF. The CSF leukocyte count was higher in patients with CNS infections [78 (IQR 6–247) vs. 5 (IQR 2–9); *p*-value: 0.002] as was protein concentration [91 (IQR 53–157) vs. 44 (IQR 30–64); *p*-value: 0.04], and the CSF-to-blood glucose ratio was lower [0.5 (IQR 0.3–0.6) vs. 0.6 (IQR 0.5–0.8); *p*-value: 0.01]. ROC analysis showed an AUC of 0.77 (95% CI 0.61–0.94) for the CSF leukocyte count and 0.75 (95% CI 0.57–0.93) for CSF protein concentration in diagnosing CNS infections. Other clinical or laboratory features, including the CD4 count, were not associated with the diagnosis of CNS infection [median CD4 count 205 (IQR 55–455) in the CNS infection group vs. 370 (IQR 150–500) in the non-CNS infection groups, *p*-value = 0.21].

The spectrum of CNS infections included PML in three (17%) cases; cryptococcal meningoencephalitis, toxoplasma encephalitis, angiostrongylus eosinophilic meningitis, and HIV encephalitis each in two (11%); and cytomegalovirus encephalitis, syphilitic meningitis, tuberculous (TB) meningoencephalitis, histoplasma encephalitis, and varicella-zoster virus (VZV) meningitis each in one (6%). Final diagnoses were microbiologically confirmed in all but two patients, which were classified diagnosed as probable viral CNS infection by the assessors. Both of them were on cART and had a CD4 count of more than 200/μl. Two patients who were diagnosed with *Angiostrongylus cantonensis* eosinophilic meningitis were young, male, and on cART and had relatively high CD4 counts (430 and 1,020/μl) and marked CSF pleocytosis (441 and 814/μl). In three (16%) patients who were diagnosed with cerebral toxoplasmosis, cryptococcal meningitis, and neurosyphilis, the CNS infection was their first manifestation of HIV infection (Tables 2, 3).

**Table 2:** Final diagnosis based on CD4 counts in HIV-infected patients and associated pathogens in episodes of central nervous system infections.

**CD4+ count/μl**	**Final diagnosis**				
	**CNS infection (53%)**	**Nervous system inflammation without infection (3%)**	**Non-infectious non-inflammatory neurological disorder (21%)**	**Non-neurological infection (21%)**	**Other systemic disorder (3%)**
<200	3 PML (23%) 2 Toxoplasma encephalitis (15%) 1 Cryptococcal meningitis (8%) 1 EBV-CMV co-infection (8%) 1 Histoplasma encephalitis (8%) 1 HIV encephalitis (9%)		2 migraine (15%)	1 pneumonia (8%) 1 systemic viral infection (8%)	1 B-cell lymphoma (8%)
200-500	2 Presumed viral meningitis/encephalitis (18%) 1 TB meningoencephalitis (9%) 1 Angiostrongylus eosinophilic meningitis (9%) 1 HIV encephalitis (9%)	1 GBS (9%)	1 toxic/metabolic encephalopathy (9%) 1 idiopathic cranial nerve palsy (9%)	1 pneumonia (9%) 1 systemic bacterial infection (9%) 1 systemic viral infection (9%)	
>500	1 Cryptococcal meningitis (13%) 1 Angiostrongylus eosinophilic meningitis (13%) 1 Syphilitic meningitis (13%) 1 VZV meningitis (13%)		2 stroke (25%)	1 leptospirosis (13%)	1 psychiatric condition (13%)

*CNS, central nervous system; PML, progressive multifocal leukoencephalopathy; EBV, Epstein–Barr virus; CMV, cytomegalovirus; TB, tuberculous; HIV, human immunodeficiency virus; VZV, varicella-zoster virus; GBS, Guillain–Barré syndrome*.

**Table 3:** HIV-infected cases with CNS infections.

	**Age, gender**	**CD4, per μL**	**cART**	**Initial presentations**	**GCS**	**Neuroimaging findings**	**CSF parameters**				**Causative pathogen**	**Diagnosis**	**Outcome, GOS**
							**Leucocyte count, per μl**	**Protein, mg/dl**	**CSF to blood Glucose ratio**	**Opening Pressure, mmHg**			
1	48, M	70	TDF, Darunavir, 3TC, Ritonavir	Fever, Bradyphrenia, dysphasic, right-sided weakness	15	Widespread non-enhancing white matter abnormalities; Multiple hypodensities in basal ganglia, internal capsule, left cerebral peduncle, and pons	6	54	0.55	–	JCV	PML	1
2	26, M	10	De novo HIV infection	H/A, fever	14	Multiple ring-enhancing lesions with central enhancing nodule and surrounding edema suggestive of toxoplasmosis	6	104	0.48	40	*Toxoplasma gondii*	Cerebral toxoplasmosis	5
3	50, M	0	None	Cranial nerve palsy, ataxia	14	Multiple hypodensities; T2/FLAIR hyperintensity in left parieto-temporal region, right thalamus, and pons	6	52	0.56	–	JCV	PML	3
4	34, M	110	De *novo* HIV infection	H/A, confusion, ataxia	11	Normal	651	206	0.41	50	*Cryptococcus neoformans*	Cryptococcal meningitis	5
5	43, M	230	Receiving cART for 2 years but discontinued it for 1 year before	H/A, nausea	14	Normal	21	81	0.98	13	HIV-1 virus	HIV-1 encephalitis	5
6	34, F	650	TDF, emtricitabine, ritonavir, atazanavir (inconsistent use)	Seizure, aphasia, attention deficit	14	Generalized edema	246	102	0.60	–	VZV	VZV encephalitis	1
7	50, M	530	TDF, emtricitabine, darunavir	H/A, nausea	15	Normal	654	180	0.22	11	*Cryptococcus neoformans*	Cryptococcal meningitis, IRIS with encephalopathy	5
8	44, M	430	TDF, 3TC, Nevirapine	Confusion, recent episode of painful skin and myalgia	13	T2 hyper intense lesions in tegmentum of pons without contrast enhancement	1,323	153	0.37	37	*Angiostrongylus cantonesis*	Angiostrongylus cantonesis eosinophilic meningitis	5
9	48, M	1,020	cART (unknown regimen)	H/A, hyperpathy in the left arm with diminished sense of vibration and position	15	Normal	2,442	91	0.37	–	*Angiostrongylus cantonesis*	Angiostrongylus cantonesis eosinophilic meningitis	5
10	37, M	330	None	H/A, nausea, bradyphrenia, fluctuating aggressive behavior	14	Normal	1,131	169	0.43	38	*Mycobacterium tuberculosis*	Tuberculous meningitis	5
11	52, M	630	De novo HIV infection	Anisocoria due to left pupillary dilatation	15	Normal	222	42	0.54	–	*Treponema pallidum*	Syphilitic meningitis and ocular syphilis	5
12	70, M	370	TDF, emtricitabine, efavirenz	Confusion, bradyphrenia, hemianopia, Babinski sign	13	Normal	27	35	0.63	15	No pathogen isolated	Mononuclear (Presumptive viral) meningoencephalitis	5
13	37, M	180	None	Fever, bradyphrenia, Babinski sign	14	Generalized edema, hypodensities in left basal ganglia	9	19	0.67	27	*Toxoplasma gondii*	Cerebral toxoplasmosis	5
14	37, F	250	3TC, ZDV, nevirapine but discontinued for 1 month before	Fever, H/A	14	Normal	546	91	0.60	39	No pathogen isolated	Mononuclear (presumptive viral) meningitis	5
15	56, M	7	TDF, emtricitabine, ritonavir	Cognitive deficits, agitation, disorientation	14	Generalized atrophy, areas of increased signal intensity in the pons and right lateral mesencephalon	81	62	0.17	10	EBV, CMV	EBV-CMV encephalitis and CMV retinitis	5
16	57, F	70	None	Episodes of confusion and disorientation, attention deficit, Babinski sign	15	Multiple hypodensities in both frontal lobes; New white matter lesions	93	63	0.70	7	JCV	PML	4
17	43, F	0	cART non-adherent	H/A, nausea, confusion	3	White matter lesions suggestive of HIV encephalitis, small intracerebral hematoma, ischemic lesions in the pons	954	216	0.27	27	*Histoplasma capsulatum*	Histoplasma capsulatum encephalitis	5
18	42, F	180	3TC, ZDV, lopinavir/ritonavir	H/A, nausea, seizure	8	White matter lesions and leptomeningeal enhancement	672	111	0.33	–	HIV-1	HIV-1 encephalitis	5

*cART, combination antiretroviral therapy; GCS, Glasgow Coma Scale; CSF, cerebrospinal fluid; GOS, Glasgow Outcome Scale; TDF, Tenofovir; 3TC, Lamivudine; JCV, John Cunningham virus; PML, progressive multifocal leukoencephalopathy; H/A, headache; ZDV, Zidovudine; EBV, Epstein–Barr virus; CMV, Cytomegalovirus*.

Overall, 8 of 34 (24%) patients had an unfavorable outcome, 4 in each group of CNS infection and other diagnoses (22 and 25%, respectively). Of these, two (11%) patients with CNS infections died. The causes of death were nosocomial sepsis and post-anoxic encephalopathy following resuscitation, and were deemed to be not related to the CNS infection.

## Discussion

Our study shows that half of HIV-infected patients with suspected CNS infections are indeed diagnosed with a CNS infection. Our population included patients with a relatively high median CD4 count (255 cells/μl), and presence of CNS infection was not associated to the CD4 count. Most of the previous studies, even after the introduction of cART, reported the spectrum of neurologic complications or CNS infections in HIV-infected patients with a median CD4 count in a range between 30 and 90 cells/μl (7, 13, 14, 16–20). A higher median CD4 count in HIV-infected patients in our study might be related to high access to HIV-related health services in the Netherlands with 89% of HIV-infected patients linked to care and 92% treated with cART in 2017 (21).

We found that CNS infections in HIV-infected patients had many different causes, of which most were opportunistic pathogens, even though the CD4 count was relatively high. This finding, despite a relatively high median CD4 count in our patients, could be partly attributed to unmasking of occult opportunistic infections as a result of immune reconstitution inflammatory syndrome (IRIS) in HIV-infected patients. Unmasking IRIS is manifested by a robust, inflammatory immune response against the infective pathogen while deteriorating the clinical condition of the patient who recently started taking cART and was previously unable to respond to the opportunistic infection because of immunosuppression (22).

The best predictor for CNS infections was the CSF leukocyte count. This is comparable with findings in HIV-negative patients with suspected CNS infections (15). Clinical characteristics had previously been found to have a low predictive value in patients with suspected CNS infections (15). Typical clinical characteristics for meningitis, such as neck stiffness, headache, fever, and an altered mental status were low among HIV patients with CNS infections (<50%). Therefore, there should be a low threshold for CSF examination in HIV-positive patients with neurological symptoms. Because of the risk of space occupying lesions, cranial imaging should be performed in these patients prior to the lumbar puncture (23). If patients have suspected bacterial meningitis, antibiotics and dexamethasone if indicated should be administered before sending them to the CT scanner (24).

We identified a broad range of causative pathogens. PML, cryptococcal meningoencephalitis, and cerebral toxoplasmosis were the most common opportunistic neurological infections. Studies from other European countries, including Spain (16), France (10), and the United Kingdom (25), also reported similar findings and identified these three opportunistic infections among the four most common causes of CNS involvement in HIV-infected patients. However, the population of HIV-infected individuals in these studies had lower CD4 counts, as compared with our cohort. Although tuberculous meningoencephalitis has been reported as one of the most frequent causes of neurological syndromes in HIV-infected patients in the countries endemic for tuberculosis (7, 12, 19, 20, 26–29), it was diagnosed in only one of our patients.

Although about one third of all patients with CNS infection in the whole cohort were diagnosed to have bacterial meningitis, none of the HIV-infected patients were diagnosed with bacterial meningitis. Previous studies on bacterial meningitis show that the incidence is 8–19 times increased in HIV-infected patients, but the absolute risk is still small (30, 31). In resource-rich countries, other causes of CNS infections have been reported to be more prevalent in HIV-infected patients (32).

Our study had several limitations. First, the number of HIV-infected patients in this study is relatively small, which makes it difficult to draw firm conclusions about the spectrum of HIV-infected patients with CNS complications. Second, only patients who underwent lumbar puncture were included in the study. As a result, we did not study HIV-infected patients whose diagnoses were confirmed by stereotactic or open surgical biopsy or were not candidate for lumbar puncture. This may influence the frequency distribution of the diagnoses because neurological disorders that manifest as single or multiple CNS mass lesions may require brain biopsy for confirmation of the diagnosis. However, recent advances in neuroimaging and molecular-based laboratory tests on CSF have increased the yield of diagnostic tests in patients with CNS infections, decreasing the need for more invasive diagnostic procedures (33).

## Conclusion

In conclusion, half of the patients with suspected CNS infections are diagnosed with a CNS infection. While the CD4 count is not a good predictor for diagnosis of CNS infection vs. other diagnosis in HIV-infected patients, CSF white cell counts are essential in making the diagnosis. Therefore, if a CNS infection is suspected, cerebrospinal fluid examination is indicated in all patients in whom lumbar puncture can be performed safely (34).

## Data Availability Statement

The raw data supporting the conclusions of this article will be made available by the authors, without undue reservation.

## Ethics Statement

The studies involving human participants were reviewed and approved by the Medical Ethical Committee of the Academic Medical Centre, Amsterdam, the Netherlands. The patients/participants provided their written informed consent to participate in this study.

## Author Contributions

FS: performed the research, analyzed the data, and wrote the first draft of the paper. FS, DB, and MB: designed the study and have read and approved the final manuscript. DB and MB: revised the paper. All authors contributed to the article and approved the submitted version.

## Funding

This work was supported by the Netherlands Organization for Health Research and Development [ZonMw; NWO-Vidi Grant (917.17.308) to MB].

## Conflict of Interest

The authors declare that the research was conducted in the absence of any commercial or financial relationships that could be construed as a potential conflict of interest.

## Publisher's Note

All claims expressed in this article are solely those of the authors and do not necessarily represent those of their affiliated organizations, or those of the publisher, the editors and the reviewers. Any product that may be evaluated in this article, or claim that may be made by its manufacturer, is not guaranteed or endorsed by the publisher.
